# 

**Published:** 1855-12

**Authors:** 


### CONTENTS.

### ORIGINAL COMMUNICATIONS.

PAGE.

ARTICLE I.—"Vis Medicatrix Naturæ and Antistasis." By JOHN W. JONES, M. D., Professor of the Principals and Practice of Medicine in the Atlanta Medical College.......... 193

ARTICLE II.—A case of Violent Uterine Hemorrhage, occurring twelve days after Delivery. By N. B. DREWRY, M. D., of Erin, Ga.......... 202

ARTICLE III.—Continuation of Report of the Medical Clinic in the Atlanta Medical College, with some general views of the Pathology and Treatment of Typhoid Fever. By J. G. WESTMORELAND, M. D., Atlanta, Ga. 204

ARTICLE IV.—Report of two Surgical Cases. By W. H. BROWN, M. D., Griffin, Georgia, Professor of Anatomy in the Atlanta Medical College.... 208

ARTICLE V.—Vicarious Menstruation. By J. BORING, M. D., Prof. of Obstetrics, &c., in the Atlanta Medical College.......... 211

### SELECTIONS.

Clinical Lectures on Hypertrophy of the Prostate with Retention of Urine.... 215

Malaria and its Cause and Effect.......... 218

Vapor of Iodine in the Treatment of Ophthalmia.......... 221

Hospital Reports—Hysterical Affection of the Hip-Joint.......... 222

Prosecution of Malpractice.......... 224

Case of Lodgement of Foreign Body in Air-Passages.......... 231

Prolapsus Uteri.......... 233

Dislocations of the Clavical.......... 236

M. Valleix.......... 241

Bristol (Mass.) South District Medical Society.......... 242

Editorial and Miscellaneous.......... 243

